# Effects of Sequence Composition, Patterning and Hydrodynamics on the Conformation and Dynamics of Intrinsically Disordered Proteins

**DOI:** 10.3390/ijms24021444

**Published:** 2023-01-11

**Authors:** Andrei Vovk, Anton Zilman

**Affiliations:** 1 Department of Physics, University of Toronto, 60 St George Street, Toronto, ON M1M 2P7, Canada; 2 Institute for Biomedical Engineering, University of Toronto, 164 College Street, Toronto, ON M5S 3G9, Canada

**Keywords:** intrinsically disordered proteins, amino acid sequence, hydrodynamic interactions, radius of gyration, end-to-end distance, sequence charge decoration, SAXS

## Abstract

Intrinsically disordered proteins (IDPs) and intrinsically disordered regions (IDRs) perform diverse functions in cellular organization, transport and signaling. Unlike the well-defined structures of the classical natively folded proteins, IDPs and IDRs dynamically span large conformational and structural ensembles. This dynamic disorder impedes the study of the relationship between the amino acid sequences of the IDPs and their spatial structures and dynamics, with different experimental techniques often offering seemingly contradictory results. Although experimental and theoretical evidence indicates that some IDP properties can be understood based on their average biophysical properties and amino acid composition, other aspects of IDP function are dictated by the specifics of the amino acid sequence. We investigate the effects of several key variables on the dimensions and the dynamics of IDPs using coarse-grained polymer models. We focus on the sequence “patchiness” informed by the sequence and biophysical properties of different classes of IDPs—and in particular FG nucleoporins of the nuclear pore complex (NPC). We show that the sequence composition and patterning are well reflected in the global conformational variables such as the radius of gyration and hydrodynamic radius, while the end-to-end distance and dynamics are highly sequence-specific. We find that in good solvent conditions highly heterogeneous sequences of IDPs can be well mapped onto averaged minimal polymer models for the purpose of prediction of the IDPs dimensions and dynamic relaxation times. The coarse-grained simulations are in a good agreement with the results of atomistic MD. We discuss the implications of these results for the interpretation of the recent experimental measurements, and for the further applications of mesoscopic models of FG nucleoporins and IDPs more broadly.

## 1. Introduction

Multiple proteins in the cell are intrinsically disordered, or possess intrinsically disordered regions that do not conform to the classical structure–function paradigm. Yet, these proteins possess various biological functions while maintaining high dynamic and structural flexibility. Under native conditions, their structures comprise dynamic ensembles of different conformations. Intrinsically disordered proteins (IDPs) or intrinsically disordered regions (IDRs) became the common nomenclature used to distinguish this class of proteins and peptides from traditional ordered proteins [1,2]. IDPs are involved in a wide range of health and disease processes and functions of the cell. Furthermore, a wide array of human diseases are associated with the failure of an ordered protein to adopt its native conformation, consequently gaining some of the properties of an IDP often resulting in aberrant aggregation [2,3]. Proteins associated with cancer, diabetes, and neurodegenerative and cardiovascular diseases often have regions of structural disorder, making them the leading targets for drug development [1,2,4].

An important example of IDPs that informs this work is the Nuclear Pore Complex (NPC), where an assembly of intrinsically disordered proteins occupies the passageway of the NPC and controls its transport properties [5]. NPC is involved a broad array of health and disease processes in the cell, and interfering with the spatial organization and dynamics of its IDPs is linked to a large number of diseases—from cancer to neurodegenerative disease. Despite substantial progress, the essential variables that dictate biophysical properties of these IDPs—known as FG nucleoporins due to the presence of characteristic FG repeats in their sequence—are still incompletely understood [5].

In another important example of IDR function, IDRs serve as linkers between different folded domains of multi-domain proteins, in signaling and other processes [6,7,8]. In some cases, the properties of such linkers can be understood based on coarse grained polymer physics models, but the effects of sequence details are still incompletely understood [8]. Precise sequences of IDRs in small linear motifs and transcription factors, among other examples, can also be important for functional specificity [9,10].

Understanding how an IDP’s amino acid sequence dictates the equilibrium and the dynamical properties of its conformational ensemble is an important step toward understanding the principles of function of this class of proteins. A full characterization of an IDP, in principle, involves a description of all possible conformational states and the rates of inter-conversion between them, which is hard to access experimentally [11]. Nevertheless, several experimental techniques reveal information about various characteristics of the IDP ensembles: NMR, fluorescence correlation spectroscopy (FCS) or dynamic light scattering (DLS) can measure the diffusion coefficient and the corresponding hydrodynamic radius of an IDP, fluorescence resonant energy transfer (FRET) provides information about the inter-residue distances (such as the end-to-end distance), and small angle X-ray scattering (SAXS) can measure the radius of gyration [2,12,13].

Emerging evidence shows that, due to their disordered nature and the importance of entropic effects, IDP structural ensembles might be less sensitive to the fine details of a specific amino acid sequence compared to the unique 3D structures of the classical folded proteins. Rather, many IDP properties can often be understood in terms of global characteristics such as the overall charge, hydrophobicity, flexibility of the polypeptide backbone and the average solvent properties [14,15,16,17,18,19]. Typically, the mean hydrophobicity is lower and the mean net charge is higher in IDP sequences than in folded proteins, and they are impoverished in large amino acids, preventing the folding of IDPs into unique stable structures with a hydrophobic core [12,20]. In one study, a predictor based on the reduction the size of the sequence alphabet by assigning each amino acid to just one of four types (neutral, hydrophobic, positive and negative), performed almost as well as a predictor using the full 20 amino acid alphabet which predicted disorder with 87% accuracy [21]. Even a minimal predictor based only on two properties: the net charge per residue and and the mean hydrophobicity per residue, can often differentiate well between IDPs and folded proteins, as well as between different classes of IDPs [1,2,3,12,20,22].

Polymer physics offers a useful theoretical framework for understanding IDP behaviors, and enables linking experimental observables to the underlying conformational ensembles [2,23,24]. Simple mean field homopolymer models have been successful in categorizing the IDP ensembles into regimes of qualitatively different behaviors based on the ensemble averages of polymer dimensions, such as the radius of gyration and the end-to-end distance [18,23,24,25]. Commonly, the size of an IDP chain in space correlates with the net balance between repulsive and attractive intra-chain and chain-solvent interactions, which can often be encapsulated in an effective internal cohesiveness parameter, related to the classical Flory parameter χ [18,20,26,27,28,29]. The ratio of the fraction of charged amino acids to the fraction of hydrophobic ones is often sufficient to distinguish between swollen and compact regimes of behavior [20,30,31].

At the low cohesiveness extreme, disordered polypeptides are often successfully described by models of polymers in a good solvent and adopt diffuse swollen random coil conformations. In the opposite, high cohesiveness, regime, the IDPs adopt dense globular conformations [23,26,32]. In particular, the location of IDPs on the order–disorder continuum can often be encapsulated in the scaling dependence of their size *R* on the chain length (number of amino acids) *N*, $R∼N^{\nu }$, which describe the universal features of the behavior of polymeric molecules that are largely independent of the details of the local microscopic properties of the chain or the solvent [1,23,25,33,34,35]. In the highly disordered regime (such as at high denaturant concentrations and low intra-chain cohesiveness), the IDP dimensions may follow the good solvent scaling law ν≃0.6, which gradually decreases to ν≃1/3 in the compact globular regime at high cohesiveness. In particular, different classes of IDPs of the NPC seem to belong to different scaling classes based on the fraction of hydrophobic residues in their sequence [18,20]. These simple mean field theories have been successful not only in describing individual molecules of IDPs but also multi-chain systems in various geometries—from surface grafted layers to 3D phase separation [18,27,28,36,37,38].

However, despite their successes, simple mean field polymer theories suffer from several drawbacks. First, they fail to differentiate between distinct polymer dimensions such as the end-to-end distance, the radius of gyration, and the hydrodynamic radius, which can lead to difficulties in the interpretation of the experimental data. Several recent works using FRET and SAXS measurements unveiled discrepancies and divergent behaviors of the different measures of polymer dimensions [39,40,41,42,43]. In particular, the chain radius of gyration $R_{g}$, inferred from FRET measurements of the end-to-end distance $R_{e}$ can show much greater compaction with the decrease in the denaturant concentration compared to the direct SAXS measurement of $R_{g}$ [39]. Similar “decoupling” between the $R_{g}$ and the end-to-end distance $R_{e}$ was observed in [43]. On the other hand Borgia et al. [41] observed consistent increase in all chain dimension with an increase in the denaturant concentration, using multiple methods: FRET for $R_{e}$, SAXS for $R_{g}$, and FCS and DLS for the hydrodynamic radius $R_{h}$. One proposed explanation for such decoupling is the effect of FRET dyes located at the chain ends [40,44,45]. On the other hand, Zheng et al. [42] and Fuertes et al. [43] did not report an observable effect of the dyes on the chain dimensions. These results raise important fundamental questions about the methodologies of inference of the chain dimensions and internal structures of IDPs from the experimental data, which may depend on the specific assumptions in the polymer models used [42,43]. The dependence of the end-to-end distance on the biophysical properties of an IDR may also play an important role in the folding and misfolding of multi-domain proteins and in the efficiency of kinase phosphorylation efficiency [6,7].

Second, simple polymer theories fail to capture the effects of sequence heterogeneity. Although some atomistic details may be successfully coarse-grained [12,24,46,47,48], the effects and the importance of the amino acid patterning on the dimensions and the dynamics of IDPs are still an area of active research [9,10,15,20,23,25,38,49,50,51,52,53,54,55]. In particular, permutations of the amino acid sequences without changing the overall composition can affect the dimensions of the polymer, as predicted computationally [25,49,50] and observed experimentally [35]. Similarly, as mentioned above, specific amino acids located near the ends of the chain might have strong effects on some of the chain properties. Furthermore, hitherto not fully explained inconsistencies arise in the measurements of the dynamic reconfiguration times of the IDPs, explored via FRET and Fluorescence Correlation Spectroscopy (FCS) [13,56,57,58].

Interpretation of the experimental data often relies on the computational models of IDPs. As mentioned above, simple mean field polymer models are powerful but often not sufficient to capture the complexity of the whole gamut of behaviors of IDPs. Computational approaches based on computer simulations offer a way to systematically study the vast sequence space and the effects of sequence heterogeneity on the polymer dimensions and other properties. All-atom molecular dynamics (MD) simulations have been used as a tool in the modeling of natively folded proteins for several decades. However, there are several obstacles when applying these methods to IDPs. Even with dramatic increases in computing power, computationally expensive simulations required to fully explore the vast conformational space of an IDP are not always feasible [11,59]. Moreover, agreed upon atomistic force fields for IDPs are still lacking, and their predictions remain sensitive to the fine-tuned choices of parameter values, and are potentially prone to overfitting [60,61,62,63].

On the other hand, coarse-grained simulations avoid many of these pitfalls by subsuming many atomistic details into the coarse-grained variables, such as local amino acid charge, hydrophobicity and monomer size [15,16,17,51,53,64,65,66]. Identification of the key properties and molecular features that capture the connection between the IDP structure and the experimentally accessible variables [65,66] while avoiding over-fitting the sparse experimental data is challenging [66]. Several of these properties have been identified: the importance of electrostatic interactions, hydrophobicity and, more generally, the association of certain amino acids with either expansion or compaction of IDPs. Yet, although a number of different force fields and solvent models have been successfully applied in different specific cases, there are currently no universally accepted coarse-grained (or atomistic) force fields. To reproduce the experimental data, simulation outcomes often require sub-ensemble sampling and re-weighting [41,43,67], or an additional ad hoc assumption about the ensemble properties [13,39,41,43,56,67].

In this paper, we systematically investigate the effects of sequence composition and heterogeneity on the dimensions and the dynamics of IDP conformational ensembles. We use experiment-informed coarse-grained minimal complexity models that include only the key features of the IDP sequence, amino acid composition and intra-chain interactions. Similar type “mesoscopic” models have been employed by us and others in applications to a number of different systems [15,38,51,53,68,69,70]. We specifically focus on the effects of the effects of chain “patchiness” and the effects of the sequence near the chain ends as compared between the homopolymer and heteropolymer models. The effects of the IDR length will be studied in the future work. The choice of the sequence properties is specifically informed by the features of FG nucleoporins known to be important for their functional properties [5]. A central feature of our analysis is the incorporation of explicit hydrodynamic effects, which are known to substantially modify polymer properties, but have so far been largely missing from the investigations of FG nucleoporins and IDPs more broadly [5].

The results shed light on the interpretation of recent experimental results and serve as a basis for further development of mesoscopic models of IDPs including those of the NPC. Furthermore, this work points out the important sequence properties that can be encapsulated in the order parameters controlling the collective multi-chain behavior such as phase separation, which has been proposed to play an important role in spatial organization of FG nucleoporins [71,72,73,74,75].

The paper is structured as follows. In Section 4, we describe the computational methods of the paper based on overdamped Langevin dynamics with explicit hydrodynamic interactions. In Section 2.1.1, we present the results of the simulations of a minimal homopolymer model of intra-chain interactions to differentiate between the various polymer dimensions: end-to-end distance, radius of gyration and hydrodynamic radius, for completeness incorporating novel and known results under the same umbrella. In Section 2.1.4, we investigate the effects of sequence heterogeneity on the IDP dimensions expanding the homopolymer model to include four monomer types (cohesive, neutral, positively charged or negatively charged).

In Section 2.2, we study the effects of the amino acid sequence on the end-to-end dynamics of IDPs and discuss the implications for the interpretation of experimental results.

We conclude with a discussion of the results and their implications for further theoretical and experimental investigations of IDPs and FG nucleoporins in particular in Section 3.

## 2. Results

### 2.1. Effects of Sequence and Interactions on the Chain Dimensions

#### 2.1.1. Effects of Internal Cohesiveness on the Chain Dimensions: Averaged Homopolymer Models

In this paper, we are specifically informed by the properties of the intrinsically disordered proteins of the nuclear pore complex, known as FG nuicleoporins (or FG nups) that owe their name to the disordered repeats of FG, FxFG and GLFG “patches” in their sequence. Hydropobic and aromatic stacking interactions between these amino acid domains result in internal cohesiveness of FG nucleoporin chains that has been suggested to play important roles in their spatial architecture and function (see, e.g., [5] for review). This internal cohesiveness is usually assessed through its effect on the chain dimensions such as the radius of gyration, end-to-end distance, hydrodynamic radius or the height extension of surface grafted chains [5]. The dependence of the IDP dimensions on their sequence is important in many other biological systems, such as inter-domain linkers in multi-domain macromolecules, small linear motifs and transcription factors [6,7,9,10].

However, several recent experiments reported discrepancies between the polymer dimensions of IDPs/chemically denatured proteins measured using different experimental techniques, most prominently FRET and SAXS [39,40]. Many of these discrepancies may result from different choices of the polymer model, the force field or the resampling procedure [41,43,67,76].

In this section, we explore the effects of the intra-chain interactions on the polymer conformational ensemble, and the corresponding experimentally relevant dimensions, such as the end-to-end distance $R_{e}$, the radius of gyration $R_{g}$ and the hydrodynamic radius $R_{h}$.

These dimensions are defined as:

(1) $$\langle R_{e}^{2}\rangle \equiv \langle {\left({\vec{R}}_{1}-{\vec{R}}_{N}\right)}^{2}\rangle$$

(2) $$\langle R_{g}^{2}\rangle \equiv \frac{1}{N}\sum_{i=1}^{N}\langle {\left({\vec{R}}_{i}-{\vec{R}}_{c}\right)}^{2}\rangle$$

(3) $$D_{e}=\frac{k_{B}T}{6\pi \eta R_{h}}$$

${\vec{R}}_{i}$ is the position of the monomer *i* and ${\vec{R}}_{c}$ is the location of the center of mass of the polymer. $D_{e}$ is the diffusion coefficient of the polymer center of mass. The Kirkwood approximation for the hydrodynamic radius is (see Supplementary Information S1):

(4) $$\langle R_{k}^{-1}\rangle \approx \frac{1}{N^{2}}\sum_{i=1}^{N}\sum_{j=1,j\neq i}^{N}\langle |{\vec{R}}_{i}-{\vec{R}}_{j}{|}^{-1}\rangle$$

In this section, we use a minimal homopolymer model which serves as a “null hypothesis” for the interpretation and analysis of the experimental data, against which more complex models can be benchmarked. In the model, all monomers of the chain interact attractively with each other with the same average interaction strength ϵ (see Equation (12)). This coarse-grained interaction parameter subsumes all the direct and solvent-mediated interactions between the monomers, solvent properties and the average composition and the sequence details of an IDP. Experimentally, low ϵ≃0 represents a protein under high denaturant conditions or an IDP with many disorder-promoting amino acids in its sequence (e.g., less cohesive FG nucleoporins such as Nsp1 [5]). Increasing ϵ represents a lower denaturant concentration or a higher fraction of order-promoting or attractive amino acids in an IDP sequence (e.g., Nup98).

The cohesiveness parameter ϵ is closely related to the classical mean field Flory interaction parameter χ [29], which encapsulates all the information about an IDP’s sequence and molecular properties; mathematically the two are related through the second virial coefficient of the interaction $\chi ≃\int d^{3}r\left(1-e^{-U(r)}\right)$, where U(r) is defined in Equations (12) and (11). Unlike mean-field models, the simulations are able to differentiate between the various polymer dimensions: end-to-end distance, radius of gyration and hydrodynamic radius.

Simulations were performed for chains of N=100 monomers and cohesive interaction strengths ranging from $\frac{\epsilon }{kT}=0$ to $\frac{\epsilon }{kT}=1.9$ inclusive, in intervals of 0.1. For each ϵ, 40 independent runs were performed, each lasting $10^{8}$ steps, with a time step of ΔT=0.001. Each run began from a self-avoiding random walk initial condition. The first $10^{6}$ steps were excluded from the analysis to avoid biasing the results by the initial conditions, and the averages were taken over the time steps and the different runs.

**Figure 1:**
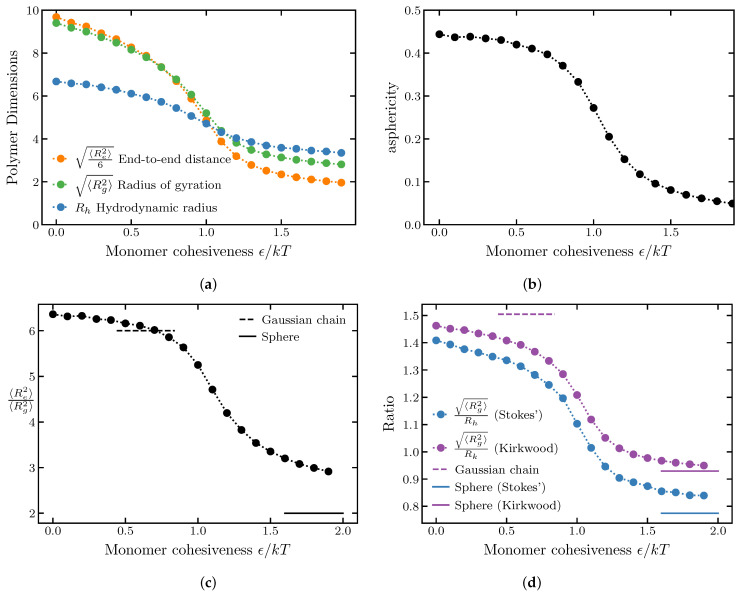
(**a**) Polymer dimensions of a homopolymer for varying monomer cohesiveness. (**b**) Asphericity of a homopolymer for varying monomer cohesiveness. (**c**) Ratio of the square of the end-to-end distance to the square of the radius of gyration of a homopolymer for varying monomer cohesiveness. The dashed lines correspond to the Gaussian chain predictions; the solid lines correspond to a uniform sphere. The ratio of square of the end-to-end distance to the square of the radius of gyration agrees with the Gaussian chain prediction ($R_{e}^{2}/R_{g}^{2}=6$) at the θ point (ϵ≈0.7−0.75kT). (**d**) Blue: ratio of the radius of gyration to the hydrodynamic radius. Purple: ratio of the radius of gyration to the Kirkwood approximation to the hydrodynamic radius. The good solvent corresponds to ϵ=0, the θ solvent corresponds to ϵ≈0.7−0.75kT and poor solvents correspond to ϵ>1.5kT. The number of monomers is N=100.

The results are summarized in Figure 1a, which shows the average end-to-end distance, the radius of gyration and the hydrodynamic radius. For presentation purposes, the end-to-end distance has been scaled down by a factor of $\sqrt{6}$ to be comparable to the other dimensions. Overall, all polymer dimensions monotonically decrease with increasing ϵ, as the chain compacts from a coil to a globule. The θ-point, where the inter-monomer repulsion is balanced by the inter-monomer attraction resulting in roughly ideal chain behavior, is located around $\frac{\epsilon }{kT}\approx 0.7-0.75$ (see Supplementary Information S2); however, the exact location of the θ-point may depend on the specific choice of the form of the interaction potential [77]. The end-to-end distance undergoes the greatest relative compaction, whereas the hydrodynamic radius experiences the least change.

One can more readily identify a polymer position on the order–disorder continuum by studying the ratios between the various polymer dimensions rather than the individual dimensions themselves in isolation. As will be seen in the next section, these ratios can be more robust and versatile measures of the polymer conformations than the individual dimensions.

Figure 1c,d show the ratios of the square of the end-to-end distance to the square of the radius of gyration, as well as the ratio of the radius of gyration to the hydrodynamic radius for varying values of the polymer cohesiveness ϵ. The ratios obtained from simulations approach the theoretical limits for good, θ, and poor solvents (calculated for N→∞). For the self-avoiding walk (ϵ=0), $\langle R_{e}^{2}\rangle /\langle R_{g}^{2}\rangle \approx 6.2-6.4$ [78,79] (depending on the approximation). For an ideal chain (θ point), $\langle R_{e}^{2}\rangle /\langle R_{g}^{2}\rangle =6$. In the compact regime of high cohesiveness, the polymer can be approximated as a uniformly dense sphere. In this regime, assuming that the locations of the two ends are independent of each other and are uniformly distributed inside the sphere, $R_{e}^{2}/R_{g}^{2}=2$ [67,80]. The ratio of the radius of gyration to the hydrodynamic radius is known to be $R_{g}/R_{k}∼1.5$ for the θ solvent [29,81] and decreases to $R_{g}/R_{h}∼0.774$ and $R_{g}/R_{k}∼0.93$ in the high-cohesiveness globular regime [67,80]. Importantly, in the homopolymer model the $R_{g}$ and $R_{e}$ remain coupled in a sense that both consistently decrease with the increase in ϵ.

#### 2.1.2. Chain Ensemble Asphericity

As mentioned above, some of the discrepancies between the FRET and SAXS measurements of the radii of gyration can be attributed to the assumptions of the homopolymer models used in the inference of polymer dimensions from the data. In particular, asphericity (sometimes referred to as the shape anisotropy) δ of IDP ensembles has been proposed to play an important role in the inference of IDP properties from FRET and SAXS data [43,67]. Although the ensemble average monomer density is isotropic for any polymer, the individual conformations may not be, giving a non-zero average asphericity. For a rigid rod, δ=1, and for a sphere δ=0. The ensemble averaged asphericity is:

(5) $$\langle \delta \rangle =1-\langle \frac{3(\lambda_{x}\lambda_{y}+\lambda_{y}\lambda_{z}+\lambda_{z}\lambda_{x})}{{\left(\lambda_{x}+\lambda_{y}+\lambda_{z}\right)}^{2}}\rangle$$

where $\lambda_{x}$, $\lambda_{y}$, and $\lambda_{z}$ are the eigenvalues of the 3×3 gyration tensor for a single conformation, whose entries are:

(6) $$S_{xy}=\frac{1}{N}\sum_{i=1}^{N}\left(R_{i,x}-R_{c,x}\right)\left(R_{i,y}-R_{c,y}\right)=\frac{1}{2N^{2}}\sum_{i=1}^{N}\sum_{j=1}^{N}\left(R_{i,x}-R_{j,x}\right)\left(R_{i,y}-R_{j,y}\right)$$

$R_{i,x}$ and $R_{c,x}$ are the *x*-components of the position of the monomer *i* and the center of mass, respectively. The radius of gyration for that conformation is: $R_{g}^{2}=\lambda_{x}+\lambda_{y}+\lambda_{z}$.

Figure 1b shows the asphericity of a homopolymer chain for different values of monomer cohesiveness and decreases from ∼0.45 for the swollen coil to close to 0 for compact globular conformations. For the homopolymer model, the asphericity is well correlated with the ratio of the end-to-end distance to the radius of gyration $R_{e}/R_{g}$.

#### 2.1.3. Conditional Sub-Ensemble Distributions

Due to the absence of universally accepted force fields to describe the conformational ensembles of the IDPs, sub-ensembles with appropriate conditional distributions of the end-to-end distance conditioned on a sub-ensembles with set values of $R_{G}$ are commonly used for comparison with the experimental data [39,67,76,82].

**Figure 2:**
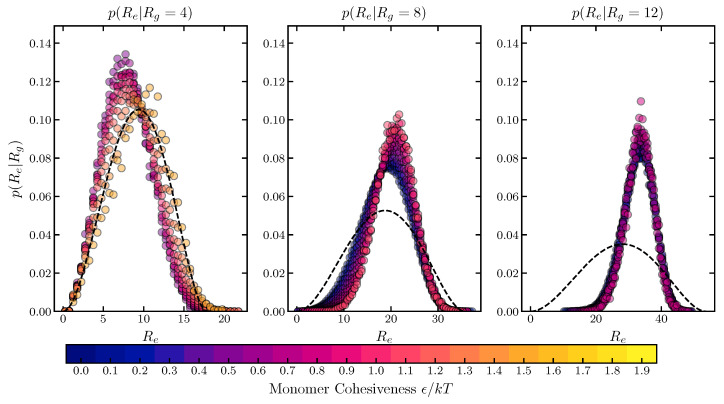
Probability distributions of the end-to-end distance of a homopolymer, conditioned on the sub-ensembles with different radii of gyration. The circle symbols show the simulation results. The color of the symbol (blue to yellow) corresponds to low to high values of ϵ. The black dashed line shows the distribution of the end-to-end distance of the Sanchez–Haran model. The number of monomers is N=100. Polymer dimensions are in the units of $\sqrt{\frac{2}{3}}b_{0}$ where $b_{0}$ is the monomer diameter. Histogram bin size for calculation of the distribution is 0.5; see Section 4.

In Figure 2, we compare the conditional distributions of the end-to-end distance, $p(R_{e}|R_{g})$, obtained from the homopolymer simulations, with the predictions of the common sub-ensemble model, Sanchez–Haran theory [82,83], which postulates that the end-to-end distance distribution of conformations conditioned on a particular radius of gyration is the probability distribution of distances between two random points inside a sphere of the radius $\sqrt{5}R_{g}$.

Notably, the simulated conditional distributions are not noticeably affected by the strength of the cohesive interaction ϵ. The Sanchez–Haran distribution matches the simulations well for compact conformations, which typically have a large ϵ, but underestimates the end-to-end distance for large conformations, which typically have a small ϵ. Thus, the Sanchez–Haran model would tend to overestimate the radius of gyration for polymers with low cohesiveness or in good solvents, based on the raw FRET data.

Another notable artifact of the Sanchez–Haran model is that it implicitly assumes that $R_{g}/R_{e}=6$ (that of a Gaussian chain) for all values or cohesiveness. Following [82]: $p\left(R_{e}\right)=\int_{R_{g,min}}^{R_{g,max}}dR_{g}p\left(R_{e}|R_{g}\right)p\left(R_{g}\right)$. Thus, $\langle R_{e}^{2}\rangle =\int_{0}^{R_{e,max}}dR_{e}R_{e}^{2}\int_{R_{g,min}}^{R_{g,max}}dR_{g}p\left(R_{e}|R_{g}\right)p\left(R_{g}\right)$$=\int_{R_{g,min}}^{R_{g,max}}dR_{g}p\left(R_{g}\right)\int_{0}^{R_{e,max}}dR_{e}R_{e}^{2}p\left(R_{e}|R_{g}\right)$. For the distribution of distances between two random points in a sphere of radius $\sqrt{5}R_{g}$, $\int_{0}^{R_{e,max}=2\sqrt{5}R_{g}}dR_{e}R_{e}^{2}p\left(R_{e}|R_{g}\right)=6R_{g}^{2}$ and so this model, like the Gaussian chain model, predicts the relationship $\langle R_{e}^{2}\rangle =6\langle R_{g}^{2}\rangle$.

These results have potentially important implications for the interpretation of the FRET and SAXS data.

#### 2.1.4. Effects of Sequence Composition and Patterning

To capture the effects of sequence composition and patterning on IDP structures, we extended the model into the heterogeneous sequence domain. In this section, rather than focusing on specific intrinsically disordered proteins with specific coarse-grained model parameters, we focus on the general relationships between the sequence properties and the polymer dimensions.

As described in Section 4, we use a “four letter” model (“HP+−”), where monomers can be either neutral/repulsive (“P”), cohesive/attractive (“H”), positively charged (“+”) or negatively charged (“−”). The first two types of monomers are inspired by the Hydrophobic–Polar model of proteins [84]. Conceptually similar mesoscopic coarse-graining has been recently used by us and others [5,38,51,52,53]. The charged monomers represent charged amino acids, while the cohesive monomers can represent order-promoting (mostly hydrophobic) amino acids, and the neutral monomers represent polar/disorder promoting amino acids. Overall, this model takes into account the basic features of IDP sequences that typically control their conformations, as the polymer dimensions are typically correlated with the compositional balance of the order-promoting and disorder-promoting amino acids [1,35,49,64]. In particular, these investigations are motivated by FG nucleoporins, where cohesive “H” type patches of different lengths are interspersed with neutral or slightly charged spacers [5].

In the model, neutral monomers experience only repulsive (non-electrostatic) interactions ($\epsilon_{i}=0$ and $q_{i}=0$ in Equations (12) and (13)). Cohesive monomers interact only with other cohesive monomers via the cohesive interaction (with strength ϵ). Charged monomers interact with other charged monomers via the electrostatic interactions, and via repulsive potentials with non-charged monomers. The bond length between adjacent monomers was 1.35 in simulation units, corresponding roughly to 0.38 nm distance between two adjacent Cα atoms in real polypeptides. For the sequences comprising mixtures of cohesive (“H”) and neutral monomers (“P”), the steric repulsion diameters of Equation (11) of all monomers were set to $B=B_{0}=\sqrt{1.5}$ in simulation units, corresponding roughly to ∼0.35 nm. For the polyampholyte sequences, the steric repulsion diameters were set to $B_{-}=2.29$ and $B_{+}=2.44$ in simulation units, reflecting the relative volumes of the corresponding amino acids (Lysine “E” and Glutamic acid “K”) [85,86] (see Section 4). The strength of the electrostatic interactions was Q=2 and the Debye length was $L_{D}=4$ in simulation units corresponding to the screening length of ∼1.1 nm (typical for ∼75 mM of NaCl). However, the results apply more generally, and we expect the small variations in the parameterization to not have a major effect on the main results of the paper.

We first investigated how the sequence patterning of neutral (“P”) and cohesive (“H”) monomers affects the chain dimensions. We simulated five different sequences of 30 cohesive (“H”) and 30 neutral (“P”) monomers using the coarse-grained model. The sequences, shown in Table 1, vary in the sizes of the cohesive and neutral clusters, increasing from 1 to 5, while maintaining the same 1:1 ratio of neutral to cohesive monomers. For each sequence and for each set of interaction parameters ϵ, *Q*, and $L_{D}$, eight runs were performed, each lasting $10^{8}$ steps, with the time step of ΔT=0.001 in simulation units. Each run began with a self-avoiding random walk initial condition. The first $10^{6}$ steps were excluded from the analysis, and the averages were taken over time and the different runs.

Specifically, we focus on the size of cohesive “patches”, which differs among the sequences while the overall composition stays the same. The “patchiness” of the sequence can be quantified using the Sequence Charge Decoration (SCD) parameter (originally introduced in [87] to describe the patterning of charged monomers). The SCD for the cohesive/neutral sequence is defined in Equation (7),

(7) $$SCD\equiv \frac{1}{N}\left[\sum_{i=2}^{N}\sum_{j=1}^{i-1}q_{i}q_{j}\sqrt{i-j}\right]$$

where *N* is the number of the monomers in the sequence, and $q_{i}=+1$ for a neutral monomer and $q_{i}=-1$ for a cohesive monomer at a position *i*.

**Figure 3:**
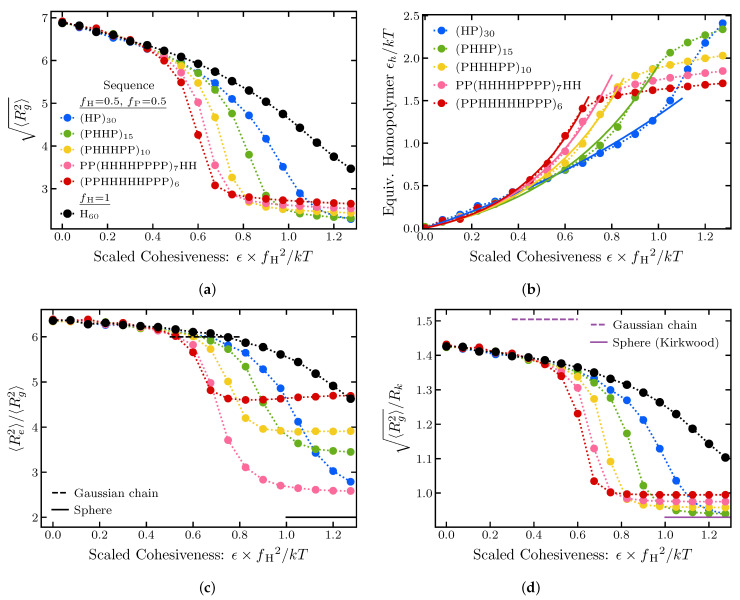
Polymer dimensions as a function of the cohesiveness. (**a**) Radius of gyration. (**b**) Equivalent Homopolymer ϵ, determined using linear interpolation. The dotted line is the equivalence to $\sqrt{\langle R_{g}^{2}\rangle }$. The solid line is a fit to $(e^{a\epsilon }-1)/b$ for the points before the inflection; see text. (**c**) Ratio of the end-to-end distance squared to the radius of gyration squared. (**d**) Ratio of the radius of gyration to hydrodynamic radius (in Kirkwood approximation). All sequences are composed of 30 cohesive and 30 neutral monomers for varying monomer cohesiveness. The size of the hydrophobic patches varies from 1 to 5; exact sequences are shown in the legend. For comparison, a homopolymer sequence of 60 cohesive monomers is shown in black. The dashed lines correspond to the Gaussian chain predictions, the solid lines correspond to a uniform sphere. *f*_H_ is the fraction of cohesive monomers in the sequence. Radius of gyration is in units of $\sqrt{\frac{2}{3}}b_{0}$ where $b_{0}$ is the monomer diameter, as described in Section 4.

The results are summarized in Figure 3, which explores the effects of the cohesiveness ϵ of the “H” monomers and the size of the cohesive “patches” on the polymer dimensions. Results for a corresponding homopolymer of 60 cohesive monomers are shown for comparison. On the *x*-axis, the monomer cohesiveness parameter ϵ is rescaled by the square fraction of cohesive monomers.

**Figure 4:**
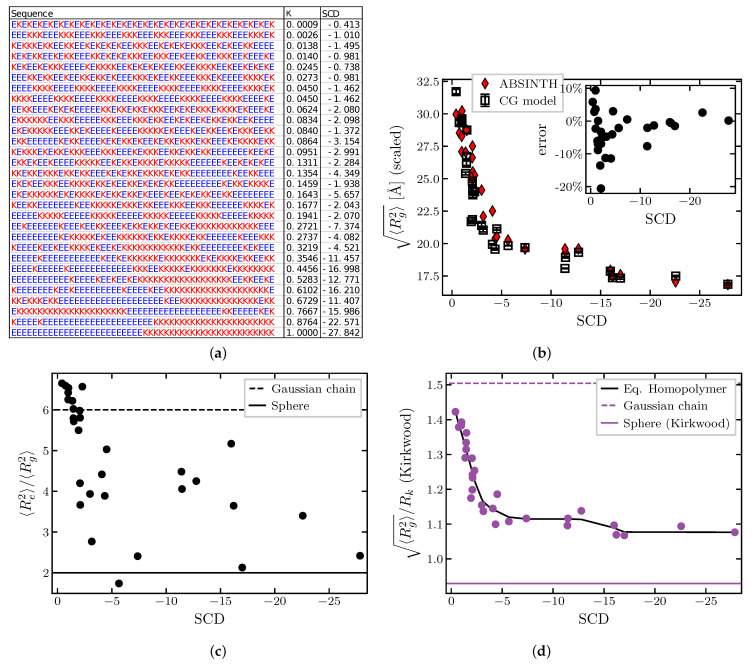
Dimensions of charged polymers. (**a**) Sequences composed of 25 positively and 25 negatively charged amino acids with their corresponding Sequence Charge Decoration (SCD) κ charge pattern parameters; see text. “K” represents positively charged lysine and “E” represents negatively charged glutamic acid. (**b**) Radii of gyration of the sequences. Black symbols: coarse-grained model; red symbols: ABSINTH model. (**c**) Squared ratio of the end-to-end distance to the radius of gyration. (**d**) Ratio of the radius of gyration to the hydrodynamic radius (in Kirkwood approximation). Solid black line is the effective homopolymer representation (see Figure 5). The dashed lines correspond to the Gaussian chain predictions, the solid lines correspond to a uniform sphere.

At low cohesiveness, the radii of gyration of all sequences collapse onto an effective homopolymer model with the corresponding value of ϵ rescaled by the fraction of cohesive monomers squared ($f_{H}=1/2$), reflecting the lower average probability of contacts between cohesive monomers in the heterogeneous sequences. The simple correspondence with the homopolymer begins to break down around ϵ≈0.4 kT. For intermediate cohesiveness, the sequences with larger “patch” sizes exhibit an earlier and steeper coil-to-globule transition. Nevertheless, as shown in Figure 3a, even moderately cohesive patchy chains can be mapped to an effective homopolymer model with effective cohesiveness that depends on the size of the cohesive patch (see also Figure 4).

Interestingly, at the high values of cohesiveness in the globular regime, the relationship between the polymer dimensions and the "patch” size is inverted: chains with larger “H” and “P” clusters have larger dimensions. This likely arises from the fact that in this regime “H” “patches” cluster to form a compact cohesive core, decorated by disordered loops of “P” containing spacers.

These trends are reproduced in the behavior of the $R_{g}/R_{h}$ ratio, as shown Figure 3d, and are even more pronounced in the ratio of the end-to-end distance to the radius of gyration (Figure 3c). These results emphasize that care must be exercised when inferring polymer properties from measurement of polymer dimensions in swollen vs. compact regimes.

We also investigated sequences containing mixtures of cohesive monomers with charges of one type (either positive or negative). Interestingly, the overall results are very similar to those of the mixtures of cohesive and neutral monomers. Essentially, in this case, charged monomers serve as neutral/repulsive monomers of a renormalized size that is dictated by the Debye length rather than the steric repulsion radius. The complete examination of this regime is outside the scope of this paper and will be presented elsewhere; see [88].

IDPs commonly contain higher fractions of both positively and negatively charged amino acids in their sequences, compared to the natively folded proteins [23,25]. In particular, FG nucleoporins are known to contain mixtures of positive and negative charges in their sequences which may play important roles in their spatial organization [5,15,16,20,54]. Importantly, for sequences with closely balanced numbers of positive and negative charges the mean field type theories usually fail due to the cancellation of the mean attractive and repulsive interactions, which necessitates in depth look into the effects of charge patterning on the IDP properties.

In particular, Das and Pappu [49] computationally investigated the effect of charge patterning on IDP properties using a family of polyampholyte sequences with different degrees of segregation of positive and negative charges in their sequences, shown in Figure 4a. Using Monte Carlo simulations of IDPs using an atomistic ABSINTH force field with implicit solvent [89], they found that the radius of gyration was higher for sequences with well mixed positive and negative charges, and lower for sequences with more segregated charge “patches”. Similar findings were obtained in both the theoretical and experimental analysis of segregation of order promoting (Proline) and charged residues [35,50].

In [49], the degree of charge segregation or “patchiness” was quantified using the parameter κ (defined in the Supplementary Information) whose value is low for well mixed sequences and high for completely segregated sequences. An alternative parameter that quantifies charge segregation and “patchiness” is known as the Sequence Charge Decoration (SCD) parameter [87], which can be defined for a polyampholyte sequence as in Equation (7) with $q_{i}=1$ for a positively charged monomer and $q_{i}=-1$ for a negatively charged one. It has been shown [90] that the radius of gyration simulated by Das and Pappu had a smoother dependence on SCD than on κ. The comparison between κ and SCD is shown in the Supplementary Information. Other conceptually similar parameters that describe the segregation of different types of monomers have been proposed in the literature as well [50].

Figure 4 shows the dependence of the various polymer dimensions on the “patchiness” of the polyampholyte sequences (quantified through SCD) calculated using the coarse-grained force field of this paper; see Supplementary Information S3 for a comparison with κ in Figure S2. As shown in Figure 4b, the coarse grained model captures well the overall compaction of the radius of gyration of the chains with the increase the charge “patch” size, as well as the sequence-specific variations in the $R_{g}$, compared to the ABSINTH model of [49]. For comparison between our results and those of Das and Pappu [49], our radii of gyration are rescaled by a factor of ∼1.4—the ratio of the average radii of gyration over all sequences between our results and those of Das and Pappu. This difference likely arises due to several assumptions of the coarse grained model that differ from the atomistic one: the bond angle restrictions between subsequent amino acids are neglected in the coarse grained model, amino acids are treated as spherically symmetric monomers ignoring the side-chain geometry, and the amino acid size in the LJ steric repulsion potential is based on the volumes of amino acids estimates in folded proteins, which could differ from the excluded volume of amino acids in IDPs [85,86]. However, most of the differences between the two models are less than 10%, as shown in the inset of Figure 4b. The sequence with the highest disagreement (approximately 20%) is with SCD=2.070, which comprises repeating periodic motifs of 5 negative amino acids followed by 5 positive ones. This particular (and biologically unlikely) sequence enables the chain to fold into an almost crystalline structure in a coarse-grained model, which is prevented by bond angle restrictions in the atomistic model.

Figure 4c,d show the ratios of the different polymer dimensions for the different sequences. Unlike the“patchy” cohesive sequences of Figure 3, for the polyampholytic sequences the ratio of the end-to-end distance to the radius of gyration is very sensitive to the specific sequence. On the other hand, the ratio of the radius of gyration to the hydrodynamic radius is correlated with SCD and the overall compaction reflected in $R_{g}$, and determines well the position of the sequence on the disorder-to-order continuum. This indicates that FRET measurements might be more indicative of local structure near the polymer ends, and cannot always used to infer the other polymer dimensions.

**Figure 5:**
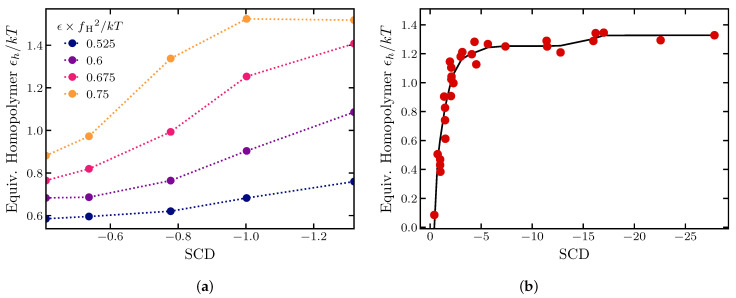
Equivalent homopolymer model. (**a**) Cohesiveness $\epsilon_{h}$ of the effective homopolymer model that reproduces the radii of gyrations of sequences with cohesiveness ϵ shown in Figure 3 and Table 1, as a function of their SCD. (**b**) Cohesiveness $\epsilon_{h}$ of the effective homopolymer model that reproduces the $R_{g}/R_{k}$ ratio of the sequences composed of 25 positively and 25 negatively charged monomers shown in Figure 4, as a function of their SCD value. The red dots show the individual correspondence for each sequence based on Figure 3. The black line is the smoothed isotonic regression $R_{g}/R_{k}$ vs. SCD; see text.

Notably, the smooth way in which the radius of gyration and the $R_{g}/R_{k}$ ratio depend on the sequence “patchiness” (SCD) resembles the dependence of the homopolymer dimensions on the cohesiveness parameter ϵ. Moreover, it has been shown [90] that SCD and $R_{g}$ are both correlated with the critical temperature of the IDP phase separation, establishing a connection between the SCD and the mean field Flory parameter χ that describes the average attraction between chain monomers [29,91]. Thus, the effect of changing the “patchiness” of a polyampholyte sequence (quantified via SCD) on the radius of gyration and the phase separation behavior of IDPs is analogous to adjusting the global average cohesiveness of the polymer. Thus, each polyampholyte sequence can be mapped onto an effective homopolymer model, by finding the homopolymer ϵ that produces the same $R_{g}/R_{k}$c ratio as the heterogeneous sequence, as shown in Figure 4d and Figure 5b.

Similar mapping can be achieved for the “HP” sequence above, as shown in Figure 3b and Figure 5c.

### 2.2. Dynamics of IDP Conformational Reconfiguration

Fluctuations in the distance between the donor and acceptor fluorophores, usually placed at the ends of the chain, result in fluctuations of the fluorescence intensity. Correlations in fluorescence intensity fluctuations, measured through the combination of FRET and fluorescence correlation spectroscopy (FCS), provide information about the internal dynamics of the chain [13,56]. The outcomes of such experiments have generated several puzzling results, and are still incompletely understood. In particular, increase in the denaturant concentration that causes swelling of the end-to-end distance, has been observed to correspond to the decrease in the end-to-end distance reconfiguration time, contrary to the naive expectation that the reconfiguration time would increase with the longer end-to-end distances [56,92]. These observations can potentially be attributed to the “internal friction” resulting from several intra-chain interactions at lower denaturant concentrations, but the physical and molecular origin of internal friction in IDPs is still under debate [13,57,58]. Theoretical approaches based on Rouse (and Zimm)-like models can capture some of the experimentally observed effects but often assume that the end-to-end distance dynamics resemble those of the end-to-end vector [93,94,95].

In this section, motivated by experimental studies of the dynamics of IDP configurational changes [56,92,96,97], we investigate the dynamics of the end-to-end distance of IDPs using several coarse-grained examples. We focus on the dynamics of the two experimentally motivated quantities: the auto-correlation times of the end-to-end-vector and the end-to-end distance.

The normalized auto-correlation function of the end-to-end vector is defined as:

(8) $$\begin{array}{c} c_{{\vec{R}}_{e}}\left(t\right)=\frac{\langle \langle {\vec{R}}_{e}\left(t\right)\cdot {\vec{R}}_{e}\left(0\right)\rangle \rangle }{\langle R_{e}^{2}\rangle } \end{array}$$

The double angle brackets represent averaging over both the initial conditions and realizations of the random simulation trajectories. The decay time of this function is referred to as the “relaxation time” of the end-to-end vector or the “rotation time” [29,93].

The normalized auto-correlation function of the end-to-end distance is defined as:

(9) $$\begin{array}{c} c_{R_{e}}\left(t\right)=\frac{\langle \langle |{\vec{R}}_{e}\left(t\right)||{\vec{R}}_{e}\left(0\right)|\rangle \rangle -\langle |{\vec{R}}_{e}{|\rangle }^{2}}{\langle {\vec{R}}_{e}^{2}\rangle -\langle |{\vec{R}}_{e}{|\rangle }^{2}} \end{array}$$

The decay time of this function is referred to as the “reconfiguration” time. It excludes contributions from the rotation modes of the entire polymer, and is closer to the reconfiguration times captured by the FRET and FCS experiments [13,93,94].

We calculate the correlation times τ of the end-to-end vector and the end-to-end distance as the integral of their normalized auto-correlation functions: $\tau =\int_{0}^{\infty }c\left(t\right)dt$ where c(t) is $c_{{\vec{R}}_{e}}\left(t\right)$ or $c_{R_{e}}\left(t\right)$ [88]. For computational convenience, the upper limit of the integral was cut off at $t=3\tau_{e}$ where $\tau_{e}$ satisfies $c\left(\tau_{e}\right)=e^{-1}$. Other methods, such as approximating the auto-correlation by an exponentially decaying function, produce substantially the same results, although further investigation is required to understand the shapes of the auto-correlation functions [88,98].

To understand the effects of sequence composition and patterning, we focus on four sequences composed of cohesive (“H”) and neutral (“P”) monomers comprising N=100 monomers each. The first sequence is the homopolymer introduced in Section 2.1.1, ${\left(H\right)}_{100}$. The second sequence consists of a repeated “HP” motif, ${\left(HP\right)}_{50}$. The two remaining sequences consist of a repeated “HPP” motif: one with cohesive monomers at the ends, ${\left(HPP\right)}_{33}H$ and the other with neutral monomers at the ends, $P{\left(HPP\right)}_{33}$.

For the homopolymer, the cohesive interactions strengths ranged from $E=\frac{\epsilon }{kT}=0$ to $E=\frac{\epsilon }{kT}=1.9$ inclusive, in intervals of 0.1. Because different heteropolymer sequences have different fractions of cohesive monomers, in order to compare end-to-end dynamics for comparable chain dimensions for the “(HP${)}_{50}$” sequence the cohesive interaction strengths were: 0.5, 1, 1.5, 2.0, 2.2, 2.4, 2.6, 2.8, 3, 3.2, 3.4, 3.6, 3.8, 4, 4.2, 4.4, 4.6, and 4.8; for the “(HPP${)}_{33}$H” and “P(HPP${)}_{33}$” sequences, the cohesive interaction strengths were: 0.5, 1, 1.5, 2, 2.5, 3, 3.5, 4, 4.5, 5, 5.1, 5.2, 5.3, 5.4, 5.5, 5.6, 5.7, 5.8, 5.9, and 6.

**Figure 6:**
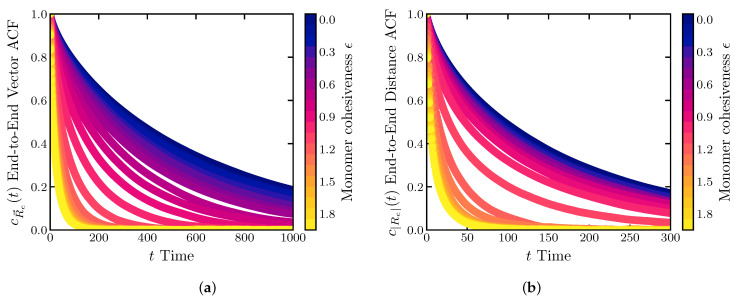
Normalized autocorrelation functions (ACF) of the (**a**) end-to-end vector and (**b**) end-to-end distance. Homopolymer model with N=100.

For each $E=\frac{\epsilon }{kT}$, 240 runs were performed, each lasting ∼$1.8\times 10^{7}$ steps, with a time step of ΔT=0.001 in simulation units. Each run began with a self-avoiding walk initial condition. The first $2\times 10^{6}$ steps were excluded from the analysis and the averages were taken both over the time and the ensemble. For each run, the auto-correlation functions were calculated using the Fast Correlation Algorithm [98]. The auto-correlation functions were subsequently averaged over different runs for each ϵ. These auto-correlation functions are shown in Figure 6.

**Figure 7:**
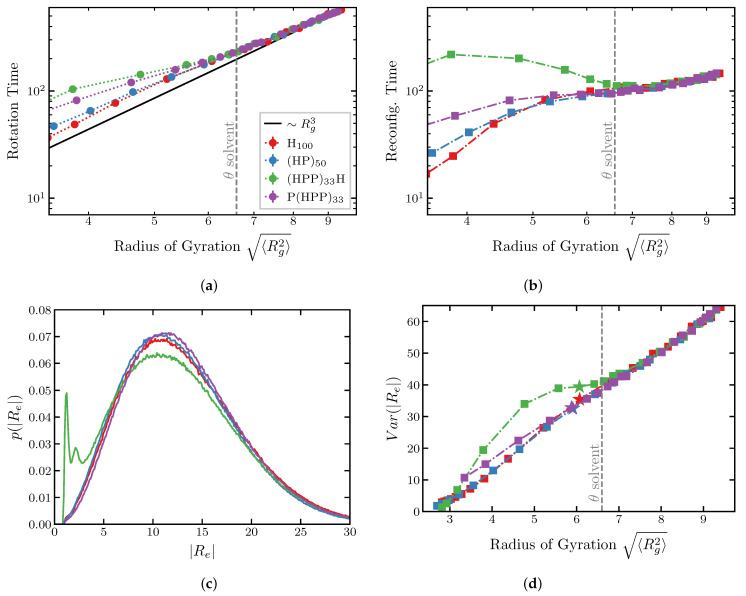
Relaxation times of the end-to-end vector and distance. (**a**) Relaxation time of the end-to-end vector (“rotation time”) and (**b**) the end-to-end distance (“reconfiguration time”) for the different sequences indicated in the legend of (**a**). The *x*-axis shows the mean square radius of gyration controlled by monomer cohesiveness in the simulations. (**c**) End-to-end distance probability distribution. Red line: H${}_{100}$ sequence; ϵ/kT=0.9. Blue line: (HP)${}_{50}$ sequence; ϵ/kT=3.2. Green line: (HPP)${}_{33}$H sequence; ϵ/kT=5.4. Purple line: P(HPP)${}_{33}$; ϵ/kT=5.6. The radius of gyration $R_{g}\approx 6\pm 0.1$ for all sequences (see (**d**)). (**d**) Variance of the end-to-end distance as a function the radius of gyration of the chains. Stars indicate the radii of gyration of the sequences for the parameter values in (**c**). Deviation of the green line from the others below the θ-point reflect the emergence of the secondary peak in the end-to-end distribution in (**c**). See text.

For all four sequences, the end-to-end vector relaxation time decreases monotonically with ϵ, Figure 7a. As expected, in the swollen regime, above the θ-point the end-to-end relaxation rotation time is well described by the classical Zimm time in the good and θ-solvent regimes $\tau_{Z}∼\frac{\eta R_{g}^{3}}{k_{B}T}∼R_{g}^{3}$, shown by the black line [29]. More globular chains below the θ-point start to deviate from the Zimm time, although agreement is still good for all sequences except (HPP)${}_{33}H$. We return to the special behavior of this sequence below.

The behavior of the end-to-end distance reconfiguration time is shown in Figure 7b. Similarly to the relaxation time of the end-to-end vector, the reconfiguration time decreases monotonically with the chain compactness for the homopolymer, the (HP${)}_{50}$ and the “P(HPP${)}_{33}$” sequences, although the dependence does not obey the Zimm law anymore. On the contrary, for the “(HPP${)}_{33}$H” sequence that has cohesive monomers at the ends, the reconfiguration time is a non-monotonic function of the chain dimensions in the compact regime below the θ-point.

This behavior can be understood by examining the distributions of the end-to-end distances for the chains of different sequences (Figure 7c). For the homopolymer, the “(HP)${}_{50}$” and the“P(HPP)${}_{33}$” sequences, the end-to-end distance distributions have a single peak around a typical value of the end-to-end-distance. However, for the “(HPP${)}_{33}$H” sequence, additional peaks emerge immediately after the polymer compacts beyond the θ solvent condition. This feature is further illustrated in Figure 7d which shows the variances of the end-to-end distance distributions for the four sequences as a function of the compactness. Above the θ-point, the variances are identical for all sequences. In contrast, below the θ-point for the “(HPP)${}_{33}$H” chain with cohesive monomers the variance is significantly higher than for the other sequences, reflecting the emergence of the secondary compact conformation shown in Figure 7c).

The transition between these two conformations—with ends bound to each other and far apart, respectively—is responsible for the non-monotonic dependence of the reconfiguration time on the chain compaction exhibited in Figure 7b). Namely, for the “(HPP${)}_{33}$H” sequence the free energy landscape in conformation space is more rugged, and the polymer is sampling a few highly probable conformations rather than smoothly transitioning between conformations of a Gaussian chain. In conclusion, the anomalous behavior of the reconfiguration time arises from the particular properties of the sequence.

These results have important implications for the interpretation of the experimental results of FRET and FCS experimental results that commonly assume a Gaussian end-to-end distribution, and where the interaction between the FRET dyes can be of importance [97]. This effect might explain the behavior observed for chemically denatured proteins and IPDs in FRET and FCS experiments [56,92,96,97].

## 3. Discussion and Experimental Implications

IDPs play important roles in many processes in the cell. One prominent example is the Nuclear Pore Complex, where the assembly of FG nucleoporins with multiple IDRs fills its transport channel and controls the transport speed, efficiency and selectivity. Although many aspects of the FG nucleoporin function can be understood within simple mean field models, disrupting specific aspects of their sequence can have profound effects on NPC architecture and function. A number of mesoscopic models that incorporate salient aspects of the FG nucleoporin sequence have been proposed but the universally accepted consensus model is still lacking.

The absence of agreed upon computational models of IDPs makes the of the experimental results difficult, and often leads to apparent discrepancies. Although specific models have been successful in explaining experimental results in a number of systems, the full picture of the effects of amino acid composition and sequence specificity on the behavior of IDPs and IDRs still remains incomplete. In this paper, we systematically investigated the effects of internal interactions and sequence heterogeneity on the dimensions of IDPs, with emphasis on the sequence “patchiness” and with potential applications to several experimental techniques. Although we use a minimal coarse-grained model, our results are likely to be general, as illustrated by their good agreement with the results obtained using atomistic simulations.

For the homopolymer model with internal cohesiveness, which serves as a “null model” against which the more complex models can be benchmarked, increase in the cohesiveness results in a consistent compaction of all polymer dimensions (end-to-end distance $R_{e}$, radius of gyration $R_{g}$ and the hydrodynamic radius $R_{h}$ (or its approximate value $R_{k}$). The degree of compaction differs for each of the polymer dimensions and their ratios ($R_{e}/R_{g}$ and $R_{g}/R_{k}$). We also found that the conformations of the homopolymers are aspherical for low values of cohesiveness, and the ratio of end-to-end distance to the radius of gyration is correlated with asphericity. In terms of dynamical quantities, both the rotation and the reconfiguration times of the end-to-end distance decreases monotonically with the polymer compactness caused by the increase in the cohesiveness. These ratios can serve as the markers for positioning the IDP in the swollen–compact continuum as a function of the internal cohesiveness (see below).

Sequence heterogeneity can significantly modulate the polymer dimensions independently of the composition or the attraction strength between cohesive monomers. For polymers composed of mixtures of cohesive and neutral monomers, an increase in the size of cohesive “patches” resulted in the more significant compaction of the polymer, reflected in all dimensions and their ratios. Nevertheless, the overall behavior of these polymers can be semi-quantitatively mapped onto that of a simple homopolymer with an appropriately chosen value of the average cohesiveness in agreement with previous works. For low values of cohesiveness below the theta-point, this effective cohesiveness is proportional to the square of the fraction of the cohesive monomers in the chain, reflecting the mean field reduction in the average number of inter-monomer contacts. For more cohesive sequences in a compact regime, the mean field description starts to break down, and the effective homopolymer cohesiveness depends on the “patch” size. In this regime, the effective cohesiveness correlates with the SCD of the sequence, which also was shown to correlate with the macroscopic Flory parameter describing the mean field cohesive behavior of single chains, and their collective properties such as the phase separation.

The presence of monomers of positive and negative charges in the sequence can have a dramatic effect on polymer dimensions, as described in Section 2.1.1. Notably, in this case the mean field description completely breaks down due to the cancellation of interactions between oppositely charged monomers. To study the effects of charge patterning, and to validate our model, we studied a polyampholyte sequence composed of positively and negatively monomers. The dimensions of the polyampholytes predicted by our coarse-grained model were similar to those predicted by an all-atom model with explicit ions, and exhibited the same trends. Overall, the radius of gyration, $R_{g}$ and the ratio of the radius of gyration to the hydrodynamic radius, $R_{g}/R_{k}$, monotonically decayed with the sequence patterning parameters SCD and κ, enabling mapping from SCD onto an average cohesiveness of an effective homopolymer model. These results are consistent with the findings that the SCD correlates with the phase transition temperature and thus with the Flory parameter χ.

However, unlike for the cohesive/neutral chains, for the polyampholytes the end-to-end distance $R_{e}$ and the ratio $R_{e}/R_{g}$ were highly sequence specific. This partial decoupling between the $R_{e}$ and $R_{g}$, arising from the high sensitivity of $R_{e}$ to the details of the sequence at the chain ends, is in agreement with previous observations and modeling. Thus, while $R_{g}/R_{k}$ ratio appears to be a robust parameter that locates the IDP on the order-disorder continuum and is useful in the interpretation of experiments, the end-to-end distance $R_{e}$ and its ratio $R_{e}/R_{g}$ are not, and care should be exercised when interpreting FRET experiments.

Nevertheless, rather than being the source of a discrepancy, the combined measurements of several polymer dimensions can guide the interpretation of experimental results and the inference of the internal interactions of an IDP. For example, the ratio between the radius of gyration and the hydrodynamic radius can reveal the location of a particular IDP on the disorder-to-order continuum, whereas the ratio of the end-to-end distance to radius of gyration may reveal the relative importance of the direct end-to-end interactions.

The sensitivity of the end-to-end distance to the properties of the monomers at the chain ends shows itself also in the end-to-end dynamics. Puzzlingly, both IDPs and chemically denatured proteins can exhibit a non-monotonic dependence of the end-to-end distance reconfiguration times on denaturant concentrations and the associated chain compaction. Molecular dynamics studies have proposed “internal friction” as the source of this behavior, but its microscopic origin still remains unclear, and the reconfiguration dynamics is still not fully understood.

The course grained model of this paper shows that the end-to-end distance distribution and thus the end-to-end distance reconfiguration time is sensitive to the properties of the monomers near the chain ends. The sequence with cohesive monomers at the ends exhibited a regime in which the reconfiguration time increases with the compaction of the polymer dimensions, qualitatively following the experimental observations. This increase was contingent on the emergence of multiple peaks in the end-to-end distance distribution due to the presence of cohesive monomers at the ends, indicating bi-stability between a compact and a swollen conformations. Chains with more homogeneous sequences explore Gaussian conformational landscapes and have faster end-to-end distance reconfiguration times, whereas those with more heterogeneous sequences explore more distant conformational states and therefore have slower reconfiguration times. This difference between the conformational ensembles would not appear in a static measurement of polymer dimensions. This emphasizes again the importance of sequence for the end-to-end dynamics and statics, and might contribute to the understanding of the origin of the “internal friction” of IDPs.

In summary, the coarse-grained models studied here encapsulate a wide range of IDP behaviors, semi-quantitatively agree with atomistic simulations, and serve as the basis for mode complex models. Our coarse-grained models add to the toolkit of computational tools for the investigation of the IDPs on mesoscopic scales and for the interpretation of the equilibrium and dynamics experiments. This study also has important limitations. In particular, it has focused on the effects of composition and sequence, but the effects of length can have important effects on IDP function, and will be systematically investigated in future work. Furthermore, in some cases, such as short linear motifs and transcription factors, the precise locations of specific residues may play an important role in conferring specificity that is not captured in the coarse grained “patchy” models. On the technical side, the employed interaction potentials do not capture potential anisotropy of some interactions, such as π−π stacking, which might be important in limiting the valence of the attractive/cohesive groups. Similarly, implementation of hydrodynamic interactions directly through the Rotne–Prager–Yamakawa formalism is relatively computationally costly and limits somewhat the lengths of the studied IDRs even with optimized coding efficiency. This can be alleviated by using dissipative particle dynamics or other coarse-grained tools to describe hydrodynamic interactions. These factors will be included, where necessary, in future investigations that will apply the coarse-grained models to specific cases of IDPs—in particular to FG nucleoporins—to understand their behavior in multi-chain assemblies and their interaction and binding with other proteins.

## 4. Materials and Methods

We represent an IDP as a polymer consisting of *N* monomers. To accommodate various levels of detail, sequence effects are introduced by assigning each monomer to one of the four types: neutral, cohesive, positively charged or negatively charged. Similar models and computational implementations have been used to represent IDPs [15,38,51,53,64,99,100,101,102,103].

The monomers are kept on a chain via the finitely extensible non-linear elastic potential (FENE) bonds between nearest neighbor monomers [104]:

(10) $$U_{FENE}=-\frac{1}{2}kl_{max}^{2}\ln\left(1-{\left(\frac{r}{l_{max}}\right)}^{2}\right)$$

All monomer pairs interact via a repulsive 8–6 LJ potential modeling the steric repulsion between the monomers:

(11) $$\begin{array}{c} U_{EV}=\left\{\begin{array}{cc} \epsilon_{LJ}\left[{\left(\frac{b}{r}\right)}^{8}-\frac{4}{3}{\left(\frac{b}{r}\right)}^{6}\right]+\frac{1}{3}\left(\epsilon_{LJ}-\epsilon \right) & \mathrm{if}\;r<b\\ 0 & \mathrm{if}\;r>b \end{array}\right.\\ b=\frac{b_{i}+b_{j}}{2} \end{array}$$

where $\epsilon_{LJ}$ is the strength of the repulsion, and *b* (equal to the sum of the radii of the two interacting monomers) is the distance between the monomer centers where the force is zero. An exception to this rule occurs if the two interacting beads are bonded monomers of a polymer: in this case $b=b_{0}$, which reflects the bond length rather than the radius. The potential is shifted by $\frac{1}{3}\left(\epsilon_{LJ}-\epsilon \right)$ in order to maintain continuity at r=b with the attractive potential described in the following section.

In addition to the universal repulsive interaction, “cohesive” monomers interact through the attractive potential

(12) $$\begin{array}{c} U_{C}=\left\{\begin{array}{cc} \epsilon \left[{\left(\frac{b}{r}\right)}^{8}-\frac{4}{3}{\left(\frac{b}{r}\right)}^{6}\right] & \mathrm{if}\;b<r<4b\\ 0 & \mathrm{if}\;r\leq b\;\mathrm{or}\;r\geq 4b \end{array}\right.\\ b=\frac{b_{i}+b_{j}}{2} \end{array}$$

The parameter ϵ controls the strength of the attraction between the monomers. The sum of the radii of the two beads (*b*) is the same as in the repulsive force described previously. The attractive potential smoothly splines with the repulsive part at r=b. To reduce computational complexity, the potential is cut off beyond r=4b, where it is ∼0.1% of its maximal depth.

Interaction between two charged monomers is modeled via the screened Coulomb potential:

(13) $$\begin{array}{c} U_{Q}=\frac{q_{1}q_{2}}{4\pi \epsilon_{0}r}e^{-\frac{r}{l_{D}}}, \end{array}$$

where $q_{1}$ and $q_{2}$ are the charges of the beads, and $\epsilon_{0}$ is the dielectric permittivity of the solution. The Debye length $l_{D}$ describes the screening of the electrostatic potential by salt ions.

The dynamics of the chain are described by the over-damped Langevin dynamics implemented via the Ermak–McCammon [105] algorithm, as described below. Hydrodynamic interactions are included via the Rotne–Prager–Yamakawa tensor [106,107].

For convenience, we define the following dimensionless variables: the position of a monomer $\vec{X}=\frac{\vec{x}}{x_{c}}$, the simulation time step $\Delta T=\frac{\Delta t}{t_{c}}$, the sum of the deterministic forces on a monomer due to its interactions with the other monomers ${\vec{F}}_{int}=\frac{{\vec{f}}_{int}}{f_{c}}$. The units of force are $f_{c}=\frac{2k_{B}T}{x_{c}}$, the units of length are $x_{c}=\sqrt{\frac{2k_{B}T}{k}}=\sqrt{\frac{2}{3}}b_{0}$ (*k* and $b_{0}$ are defined below), and the units of time are $t_{c}=\frac{\xi_{0}}{k}$, where $\xi_{0}=6\pi \eta a_{0}$, is the Stokes drag coefficient for a bead with hydrodynamic radius $a_{0}$. In these units, the displacement of a monomer in one simulation time step is:

(14) $$\Delta \vec{X}=\bar{M}{\vec{F}}_{int}\Delta T+\bar{H}\vec{\Delta W}.$$

When hydrodynamic interactions are included, $\bar{M}$ is the Rotne–Prager–Yamakawa tensor [107] multiplied by $\xi_{0}$, and $\bar{H}{\bar{H}}^{⊺}=\bar{M}$. In the simulations, $\bar{H}$ is chosen to be a lower triangular matrix obtained using the Cholesky decomposition of $\bar{H}$. For the calculation of the equilibrium quantities, such as the radius of gyration of the end-to-end distance, hydrodynamic interactions are immaterial and all off-diagonal entries of $\bar{M}$ can be set to 0. The components $\Delta W_{i}$ of $\vec{\Delta W}$ are independent random variables with Gaussian distributions such that $\langle \Delta W_{i}\rangle =0$ and $\langle \Delta W_{i}\left(T\right)\Delta W_{j}\left(T^{\prime }\right)\rangle =\Delta T\delta \left(T^{\prime }-T\right)\delta_{ij}$ [105,108,109]; see Supplementary Information for details.

Expressed in the simulation units, the range (diameters) of the repulsive volume interactions between the bonded monomers is $B_{0}=\frac{b_{0}}{x_{c}}=\sqrt{\frac{3}{2}}$. The maximal extension of the FENE bonds between monomers is $L_{max}=\frac{l_{max}}{x_{c}}=2B_{0}$. The strength of the excluded volume interactions is $\frac{\epsilon_{LJ}}{kT}=1$. The hydrodynamic radii of the monomers are $A=\frac{a}{x_{c}}=\frac{b}{2x_{c}}=\frac{B}{2}$.

## Figures and Tables

**Table 1:** Sequences composed of 30 cohesive monomers (“H”) and 30 neutral monomers (“P”) of different sizes (1, 2, 3, 4 or 5) of cohesive (and neutral) clusters. The subscripts indicate how many times the sequence in parentheses is repeated.

Sequence	SCD
(HP${)}_{30}$	−0.410
(PHHP${)}_{15}$	−0.537
(PHHHPP${)}_{10}$	−0.778
PP(HHHHPPPP${)}_{7}$HH	−1.002
(PPHHHHHPPP${)}_{6}$	−1.319

## Data Availability

The simulation code used in generation of the data presented in the figures can be found at https://github.com/aivovk/IJMS-IDP-dimensions accessed on 3 November 2022.

## Supplementary Materials

The following supporting information can be downloaded at: https://www.mdpi.com/article/10.3390/ijms24021444/s1. References are cited in [110,111,112].
