# Supplementary material for: Effects of Sequence Composition, Patterning and Hydrodynamics on the Conformation and Dynamics of Intrinsically Disordered Proteins
Source: Int J Mol Sci. 2023 Jan 11;24(2):1444. doi: 10.3390/ijms24021444 (PMC9867189; doi:10.3390/ijms24021444)
Supplement: Supplementary file 1 [file ijms-24-01444-s001.zip › ijms-2045665-supplementary.pdf]

Supplementary Information for: Effects of Sequence Composition, Patterning and Hydrodynamics on the Conformation and Dynamics of Intrinsically Disordered Proteins

S1 Hydrodynamic Radius and the Kirkwood Approximation

The hydrodynamic radius R_h is the radius of a solid sphere that has the same diffusion coefficient as the polymer chain. It depends not only on the equilibrium properties of the conformational ensemble but also on the dynamical intra-chain correlations mediated via fluid flow around and within the chain. The diffusion coefficient of a polymer chain is inversely proportional to the hydrodynamic radius via the Stokes-Einstein relation [1, 2]:

$$D_e = \frac{k_B T}{6\pi\eta R_h} \tag{S1}$$

where k_B is Boltzmann's constant, T is the absolute temperature, η is the solvent viscosity. D_e can be calculated from displacement of the polymer's centre of mass as [3, 4]:

$$D_e = \lim_{t\to\infty} \frac{\langle (\vec{R}_c(t) - \vec{R}_c(0))^2 \rangle}{6t}, \tag{S2}$$

where the average is over ensemble trajectories and initial conditions.

Kirkwood and Riseman [5] introduced a pre-averaging approximation for the hydrodynamic interactions between the monomers, allowing to calculate the approximate hydrodynamic radius from just the equilibrum ensemble of conformations. The Kirkwood approximation for the diffusion coefficient of a polymer, using the Oseen tensor for hydrodynamic interactions, is [3]:

$$D_k = \frac{k_B T}{6\pi\eta N^2} \left(\sum_{i=1}^{N} \left(\frac{1}{a_i} + \sum_{j=1, j\neq i}^{N} \langle |\vec{R}_i - \vec{R}_j|^{-1} \rangle \right) \right) \tag{S3}$$

Here, a_i is the hydrodynamic radius of a monomer i, and the average is over the equilibrium ensemble of conformations. The inverse of the approximation to the hydrodynamic radius is defined as:

$$\langle R_k^{-1} \rangle = \frac{1}{N^2} \left(\sum_{i=1}^{N} \left(\frac{1}{a_i} + \sum_{j=1, j\neq i}^{N} \langle |\vec{R}_i - \vec{R}_j|^{-1} \rangle \right) \right) \tag{S4}$$

Using Brownian dynamics simulations with implicit hydrodynamic interactions, Liu et al. [3] and Schmidt et al. [6] have previously found that the Kirkwood approximation overestimates the hydrodynamic radius by $< 4\%$ for a SAW and a worm-like chain model. In this paper, we extended the comparison between the Kirkwood approximation and the hydrodynamic radius to all values of cohesiveness. The Kirkwood approximation overestimates the true diffusion coefficient by 3-5% in agreement with other studies [3, 6]. In the poor solvent regime the relative difference increases to beyond 10% and is larger for longer polymers (Fig. 1d).

S2 Scaling Exponent of Radius of Gyration

We investigated the effects of monomer cohesiveness ϵ on the scaling exponent ν of the radius of gyration of a homopolymer with the number of bonds N: $\langle R_g^2 \rangle \propto N^{2\nu}$.

We performed simulations of homopolymers with $N+1 = 50, 100, 150, 200, 300, 400$ monomers at $\epsilon = 0, 0.64, 0.7, 0.75$ kT. All other simulation parameters were the same as the homopolymer model of Section 2. The total runtime (number of steps) and number of independent runs (from different initial conditions) varied. Simulations where initialized from a self-avoiding walk ($N+1 = 50, 100, 150, 200$) or a random walk initial condition ($N = 200, 300, 400$). Correlation functions of $R_g^2(t)$ were calculated for $N+1 = 50, 100, 150$ and fit with exponential decays in order to estimate the correlation times τ_N. The initial $2\tau_N$ or more of each simulation were excluded from the analysis. The error bars were estimated as $\mathrm{Var}(\langle R_g^2 \rangle) = \frac{\mathrm{Var}(R_g^2)}{n_s n_t}$ where n_s is the number of independent runs and $n_t = \frac{t_r}{2\tau_N}$, where t_r is the minimum simulation time included in the analysis for that N [7].

Figure S1 shows the dependence of the radius of gyration on the number of bonds N for different monomer cohesiveness ϵ; For $\epsilon = 0$, as expected, the chain behaves as a self-avoiding random walk in a god solvent with $\nu = 0.588 \pm 0.001$ [2]. The θ-point, where $\nu = 0.5$, is located between $\epsilon = 0.74$ kT and 0.75 kT. This is consistent with the second virial coefficient for the inter-monomer interaction being 0 at $\epsilon \approx 0.64$ kT. The exact location of the θ-point depends on the details of the repulsive and attractive potentials used in the model [8].

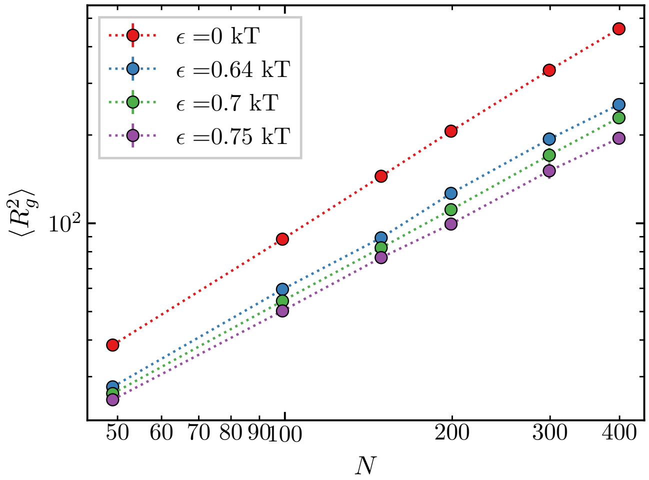

(a)

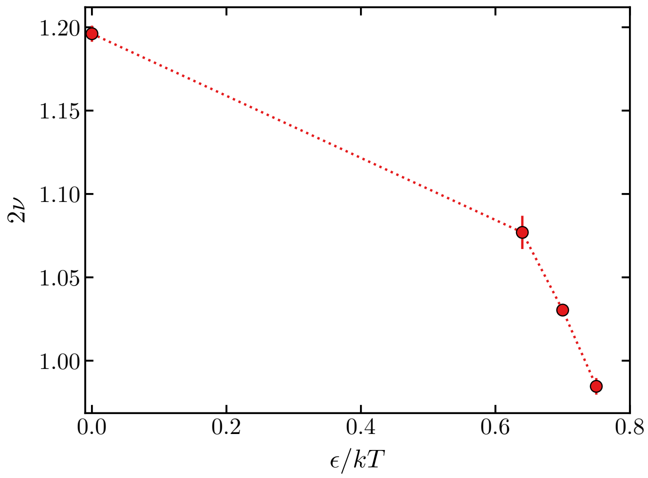

(b)

Figure S1: (a) Dependence of the radius of gyration on the number of bonds in the chain for different monomer cohesiveness ϵ. (b) The variation of the scaling exponent of $\langle R_g^2 \rangle \propto N^{2\nu}$ with monomer cohesiveness ϵ.

S3 Comparison between sequence patterning parameter SCD and κ.

Figure S2 shows the polymer dimensions and the ratios of dimensions of polyampholyte sequences plotted against the charge sequence parameter κ introduced by Das and Pappu [9].

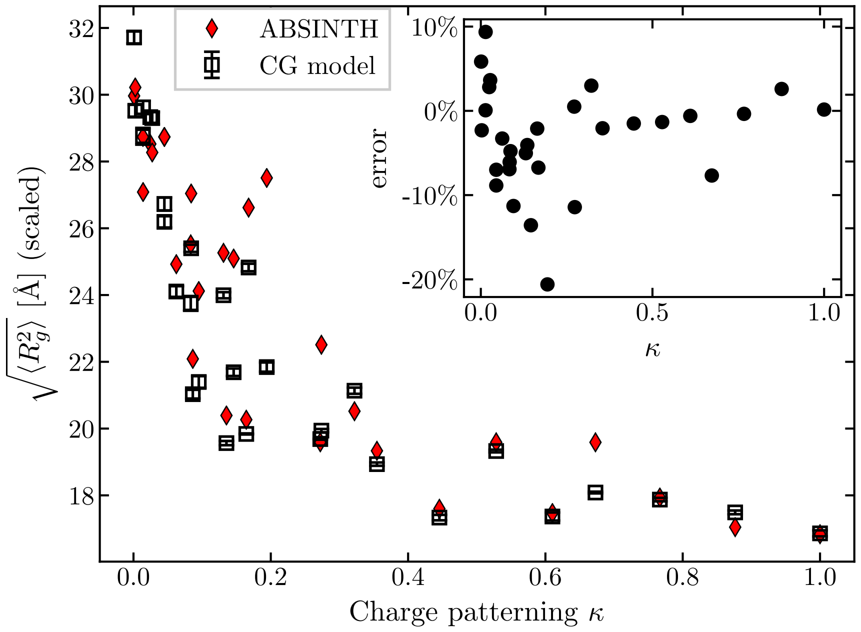

(a)

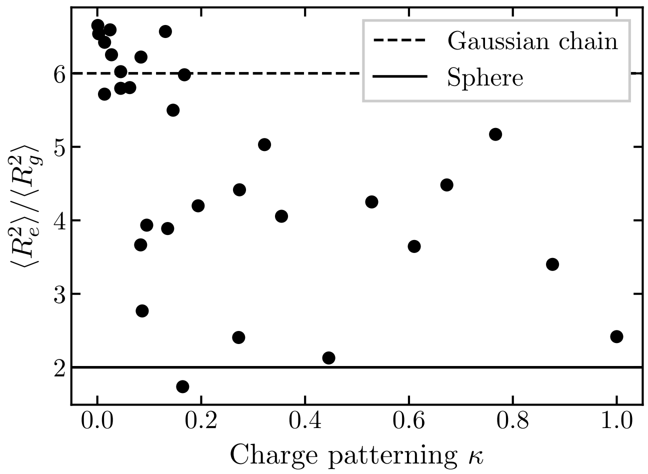

(b)

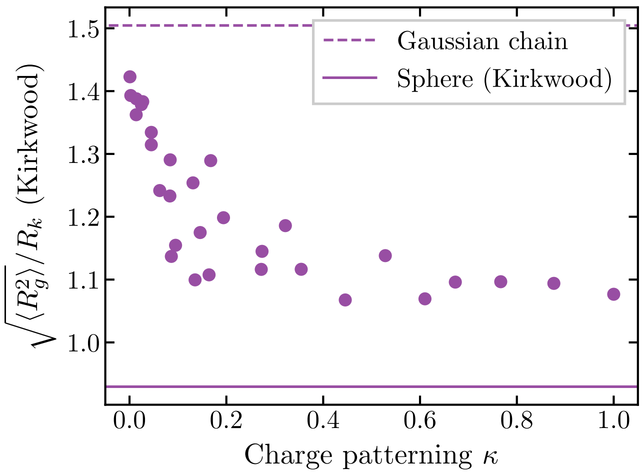

(c)

Figure S2: Dimensions of polymers composed of 25 positively and 25 negatively charged monomers. Same as Figure 4 but plotted with the κ parameter introduced by Das and Pappu [9] on the x-axis. *(a)* Radius of gyration compared with ABSINTH model. *(b)* Square end-to-end distance to square radius of gyration. *(c)* Radius of gyration to hydrodynamic radius (Kirkwood approximation). The dashed lines correspond to the Gaussian chain predictions, the solid lines correspond to a uniform sphere. The full sequences and their corresponding κ and SCD parameters are shown in Fig. 4a.

References

[1]P. G. De Gennes, *Scaling concepts in polymer physics* (Cornell university press, Ithaca N.Y., 1979).

[2]M. Doi and S. F. Edwards, *The Theory of Polymer Dynamics* (Clarendon Press, Oxford, 1986).

[3]B. Liu and B. Dünweg, "Translational diffusion of polymer chains with excluded volume and hydrodynamic interactions by Brownian dynamics simulation", The Journal of Chemical Physics **118**, 5057 (2003).

[4]R. Rodríguez Schmidt, J. G. Hernández Cifre, and J. García de la Torre, "Translational diffusion coefficients of macromolecules.", European Physical Journal E, Soft matter **35**, 9806 (2012).

[5]J. G. Kirkwood and J. Riseman, "The intrinsic viscosities and diffusion constants of flexible macromolecules in solution", The Journal of Chemical Physics **16**, 565–573 (1948).

[6]R. R. Schmidt, J. G. Cifre, and J. G. De La Torre, "Comparison of Brownian dynamics algorithms with hydrodynamic interaction", Journal of Chemical Physics **135**, 84116 (2011).

[7]D. Frenkel and B. Smit, *Understanding molecular simulation : from algorithms to applications* (Academic Pres, 2002), p. 638.

[8]M. O. Steinhauser, "A molecular dynamics study on universal properties of polymer chains in different solvent qualities. Part I. A review of linear chain properties", Journal of Chemical Physics **122**, 94901 (2005).

[9]R. K. Das and R. V. Pappu, "Conformations of intrinsically disordered proteins are influenced by linear sequence distributions of oppositely charged residues", Proceedings of the National Academy of Sciences **110**, 13392–13397 (2013).
